# Vitamin D3 Supplementation Attenuates Surgery-Induced Neuroinflammation and Cognitive Impairment by Regulating NLRP3 Inflammasome in Mice

**DOI:** 10.1155/2022/4696415

**Published:** 2022-11-29

**Authors:** Jingyan Jiang, Xin Huang, Xiang Gao, Shenghui Yu

**Affiliations:** Department of Anesthesiology, The Affiliated People's Hospital of Ningbo University, Ningbo 315040, China

## Abstract

Neuroinflammation plays a dominant role in the progression of postoperative cognitive dysfunction (POCD). Vitamin D has been known to have important regulatory functions in inflammation and immune response. The NOD-like receptor protein 3 (NLRP3) is an essential inflammasome in the inflammatory response and could be activated by anesthesia and surgery. In this study, male C57BL/6 mice aged 14–16 months were given VD3 for 14 days straight before having an open tibial fracture surgery. The animals were either sacrificed to obtain the hippocampus or tested in a Morris water maze test. Western blot was employed to estimate the levels of NLRP3, ASC, and caspase-1, immunohistochemistry was used to identify microglial activation, and an enzyme-linked immunosorbent assay was used to measure the expression of IL-18 and IL-1*β*, while using the corresponding assay kits to assess ROS and MDA levels to reflect the oxidative stress status. We showed that VD3 pretreatment significantly improved surgery-induced memory and cognitive dysfunctions in aged mice, which was linked to the inactivation of the NLRP3 inflammasome and the inhibition of neuroinflammation. This finding provided a novel preventative strategy for clinically reducing postoperative cognitive impairment in elderly surgical patients. This study has some limitations. Gender differences in the effects of VD3 were not considered, and only male mice were used. Additionally, VD3 was given as a preventative measure; however, it is unknown whether it has any therapeutic benefits for POCD mice. This trial is registered with ChiCTR-ROC-17010610.

## 1. Introduction

Postoperative cognitive dysfunction (POCD) is a clinical condition that occurs following anesthesia and surgery in patients, particularly in the elderly [1]. Clinical studies have provided evidence that POCD may be linked to increased mortality, poorer quality of life, and prolonged hospitalization [1, 2]. However, the pathophysiology of POCD remains unknown.

Vitamin D deficiency has emerged as a global public health issue, especially for the elderly [3, 4]. There are limited investigations on the connection between vitamin D levels and POCD in elderly patients. A recent prospective cohort study found that vitamin D deficiency can increase the risk of neurocognitive disorders in elderly individuals during the perioperative period, presumably because low vitamin D levels cannot effectively prevent the postoperative oxidative stress increase [5]. In addition, the supplementation of the active vitamin D metabolite 1,25-dihydroxy vitamin D3 (1,25(OH)_2_D3, calcitriol, VD3) provided protective effects on memory and learning in animal models of LPS, hepatectomy, and sevoflurane anesthesia-induced cognitive dysfunction [6–8]. Therefore, vitamin D may be developed as a potential therapy for POCD; however, the probable mechanism is still unknown.

Accumulating evidence from clinical and experimental studies has shown that POCD is driven by neuroinflammation-related proinflammatory cytokines [9]. NLR pyrin domain-containing 3 (NLRP3) activation has recently been widely explored and is associated with neuroinflammation [9–12]. NLRP3 assembles a multiprotein complex (inflammasome) consisting of NLRP3, apoptosis-associated speck-like protein containing a caspase recruitment domain (ASC), and caspase-1 [13]. Activation of inflammasomes can promote the production and release of proinflammatory cytokines such as IL-1*β* and IL-18. Numerous studies have demonstrated that VD3 inhibits the NLRP3 inflammasome, which protects against various inflammatory diseases. Duan et al. [14] found that VD3 targeting to vitamin D receptor prevents chondrogenic extracellular matrix degradation through regulating macrophage NLRP3 activation and inflammatory cytokines secretion, thereby alleviating osteoarthritis. Dong et al. [15] found that VD3 ameliorated nitrogen mustard-induced cutaneous inflammation by inactivating the NLRP3 inflammasome.

Based on the above evidence, this study is aimed at determining whether VD3 attenuated surgery-induced cognitive impairment by modulating hippocampal neuroinflammation through the inactivation of the NLRP3 inflammasome.

## 2. Materials and Methods

### 2.1. Animals

Male C57BL/6 aged 14-16 months old was purchased from Beijing Vital River Laboratory Animal Technology (China). The mice were housed under specific pathogen-free conditions (ambient temperature, 22.0 ± 1.0°C, and humidity, 40%) during breeding and the experiments. Food and water were available *ad libitum*. All experimental procedures involving animals were approved by the Animal Care and Use Committee of Ningbo University in following guidelines for the Care and Use of Laboratory Animals by the National Institutes of Health.

**Figure 1:**
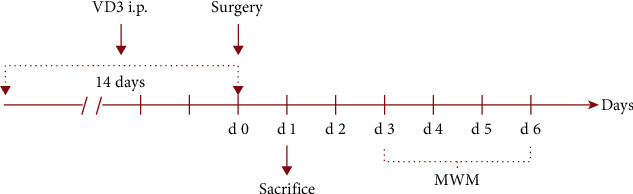
Experimental design. MWM: Morris water maze.

The mice were randomly assigned to one of four groups: control (con), surgery (sur), surgery + VD3 (sur + VD3), and surgery + vehicle (sur + veh). Half of the animals in each group underwent behavioral testing while the other half were sacrificed 24 hours following the surgery for molecular detection to minimize the potential confounding effects of behavioral tests on inflammatory markers (Figure 1).

### 2.2. Surgical Trauma

We performed open tibial fracture surgery as previously described [16–18]. The left hind paw was shaved, disinfected, and cut open to strip the periosteum, and an osteotomy was performed. Then, a 0.38 mm pin was inserted into the intramedullary canal. After this fracture and fixation, the wound was irrigated with povidone-iodine, and the skin was sutured with 4/0 Prolene sutures.

The anesthetics are established by employing a sealed chamber to give 3-5% sevoflurane with 100% oxygen, followed by a mouse anesthesia mask attached to an animal anesthesia equipment to maintain 2-3% sevoflurane-O2 (R500SE, RWD Life Science, Shenzhen, China).

### 2.3. Morris Water Maze (MWM)

A water-filled circular tank was surrounded by visual cues and divided into 4 quadrants, with a platform below the water surface in the target quadrant. Acquisition started on postoperative day 3, and a probe trial test was conducted on postoperative day 6. During the acquisition session, a trial was terminated when the mice reached the platform, where they were allowed to stay for 15 seconds. The animal was manually taken to the platform and kept there for 15 seconds if it cannot find the platform within 60 seconds. During the probe test, the platform was removed, and the search behavior was tracked again. A video tracking system was used to analyze the moving paths and the amount of time required to locate the quadrant or the hidden escape platform (EthoVision XT, Noldus Instruments, Wageningen, Holland).

### 2.4. Treatment with VD3

The animals were injected with VD3 (Sigma-Aldrich Co., St. Louis, MO, USA) intraperitoneally every day starting on day 14 before surgery (VD3 dose: 2.5 ng/g body weight). Sterile corn oil served as the vehicle for the VD3 solution [8].

When mice were sacrificed, 0.5 ml of blood was collected, and after standing for 30 min, it was centrifuged at 3,000 r/min at 4°C for 20 min, and the supernatant serum was collected to detect the content of VD (25(OH)D) by ELISA.

### 2.5. Western Blotting

The hippocampus was collected and homogenized in a lysis buffer containing protease inhibitors (Thermo Scientific, Carlsbad, USA). Samples were separated on 10% SDS-PAGE gels and transferred to a PVDF membrane (0.22 *μ*m; Millipore, Temecula, CA, USA). The membrane was blocked with 5% nonfat dry milk in Tris-buffered saline and then incubated overnight with NLRP3 (1 : 1000; CST, USA), ASC (1 : 1000; CST, USA), caspase-1 p20 (1 : 500, Santa Cruz, CA), and GAPDH (1 : 5000; Proteintech, China). After washing, the membrane was then incubated with 800 CW goat anti-rabbit IgG antibody (1 : 15000, LI-COR, Nebraska, USA) or 800 CW goat anti-mouse IgG antibody (1 : 15000, LI-COR, Nebraska, USA) for 60 minutes. Target bands were revealed with a fluorescence scanner (Odyssey Infrared Imaging System, LI-COR Biotechnology, NE, USA). To quantify the bands, western blots were analyzed using the ImageJ analysis software (NIH, USA). Protein band volumes were assessed, and protein band values were standardized by GAPDH values from the same samples.

### 2.6. Enzyme-Linked Immunosorbent Assay (ELISA)

The mice were anesthetized with sevoflurane before being decapitated, and the brain was quickly removed and dissected to collect the hippocampus. Interleukin-1*β* (ExCell Bio, Taicang, China) and interleukin-18 (R&D Systems, Minneapolis, USA) were measured using ELISA after the samples had been rinsed with cold saline solution. The preparation of all reagents, the working standards, and the protocol was followed according to the manufacturer's instructions.

### 2.7. Immunohistochemistry

After anesthesia, mice were transcardially perfused with a saline solution followed by 4% paraformaldehyde (PFA). The brain was then dissected out, fixed with 4% PFA overnight, and consecutively incubated for 24 hours each in 15% and 30% sucrose solutions. The brain was then frozen in an optimal cutting temperature compound and cut into 25 *μ*m thick sections (CM1950, Leica, Frankfurt, Germany). Sections containing the hippocampus were incubated overnight at 4°C in 0.1 M PBS buffer containing 0.5% Triton X-100 and goat anti-ionized calcium-binding adapter molecule 1 (Iba-1, 1 : 500; Abcam, Cambridge, USA). After that, sections were washed three times in PBS solution and then incubated for 90 minutes at room temperature in the same PBS solution containing Alexa 488-conjugated donkey anti-goat antibody (1 : 500; Abcam, Cambridge, USA). Three sections were imaged per mouse using a confocal laser scanning microscope (SP8, Leica, Frankfurt, Germany). Iba-1 staining was analyzed in a blinded manner using the ImageJ software (NIH, USA). The number of pixels per image with intensity above a predetermined threshold level was considered to be positively stained. The degree of positive immunoreactivity was reflected by the percentage of the positively stained area in the total area of the interested structure in the imaged field.

### 2.8. Oxidative Stress Detection

To detect reactive oxygen species (ROS) in the hippocampus of mice, the ROS Assay Kit (Nanjing Jiancheng Bioengineering Institute, China) was employed. After collecting the supernatant, the protein concentration was determined. One hundred ninety microliters of supernatant was added to ten liters of 2,7-dichlorofluorescin diacetate, and samples were incubated at 37°C for 30 minutes. The results were expressed as fluorescence intensity/100 mg protein after the fluorescence was measured (excitation wavelength of 500 nm and emission wavelength of 530 nm).

The malondialdehyde (MDA) levels in the hippocampus of mice were measured using an MDA Assay Kit. Following the protein quantification, the appropriate reagent was added in accordance with the manufacturer's instructions to create the reaction mixture, which was then heated at 100°C for 20 minutes before being centrifuged at 3500 g for 10 minutes at room temperature. The absorbance was measured at 532 nm. The data were given in nanomoles per milligram (nmol/mg) of protein.

### 2.9. Statistical Analysis

Statistical analysis was performed using GraphPad Prism 8.0 (GraphPad Software, San Diego, USA). All data are expressed as mean ± standard deviation (SD). Statistical comparisons were performed using a one-way analysis of variance (ANOVA) followed by Bonferroni's post hoc test. *P* < 0.05 was considered statistically significant.

## 3. Results

### 3.1. VD3 Treatment Improved the Cognitive Impairment of Aged POCD Mice

**Figure 2:**
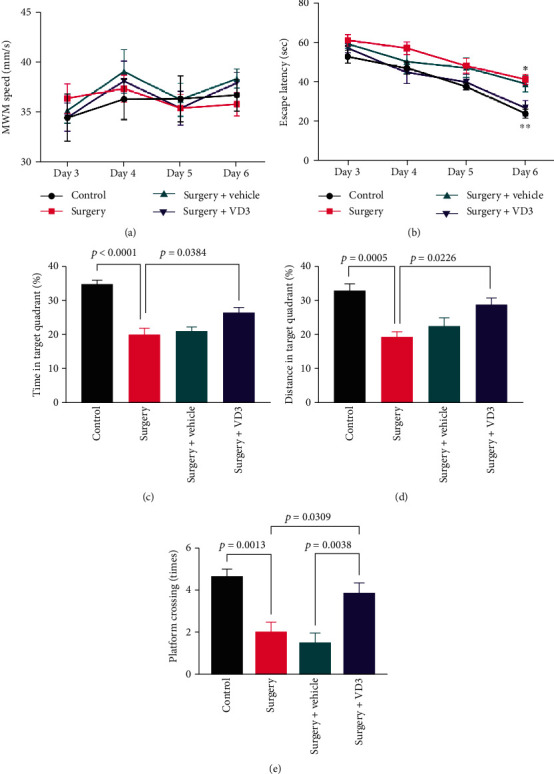
The effect of VD3 on surgery-induced postoperative cognitive impairment in aged mice. (a) Swimming speed of mice in the MWM test. (b) Escape latency in the MWM test. (c, d) Time and distance in the target quadrant. (e) Numbers of platform crossing. Data are presented as mean ± SD (*n* = 8 per group).

The spatial memory was evaluated using the Morris water maze test. Tibial fracture surgery caused animals to have significantly longer escape latency during the acquisition procedure on day 6 (*t* = 5.052, *P* < 0.05, Figure 2(b)). In comparison to the surgery group, the mice administered with VD3 experienced a reduced escape latency (*t* = 3.152, *P* < 0.01, Figure 2(b)). In the probe test, the number of platform crosses was less in the surgery group than in the control group (*t* = 4.249, *P* < 0.01, Figure 2(e)), but it was higher in the VD3-treated mice (*t* = 3.035, *P* < 0.05, Figure 2(e)). Furthermore, VD3-treated mice traveled farther and spent more time in the target quadrant (*t* = 2.947, 3.161, both *P* < 0.05, Figures 2(c) and 2(d)). There was no significant change in swimming speed between groups (*F* = 0.657, *P* > 0.05, Figure 2(a)), proving that mice with tibial fracture surgery had an unaffected motor function. Overall, VD3 repaired neurological deficits in memory and learning induced by surgery.

### 3.2. VD3 Inhibited Surgery-Induced NLRP3 Inflammasome Activation in the Hippocampus of Aged Mice

**Figure 3:**
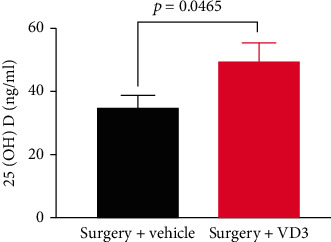
Serum 25(OH)D content following surgery. Data are presented as means ± SD (*n* = 6 per group).

The ELISA was employed to measure the serum 25(OH)D level. The results demonstrated that the mice receiving the VD3 treatment exhibited greater 25(OH)D concentrations than the vehicle group (*P* < 0.05) (Figure 3).

**Figure 4:**
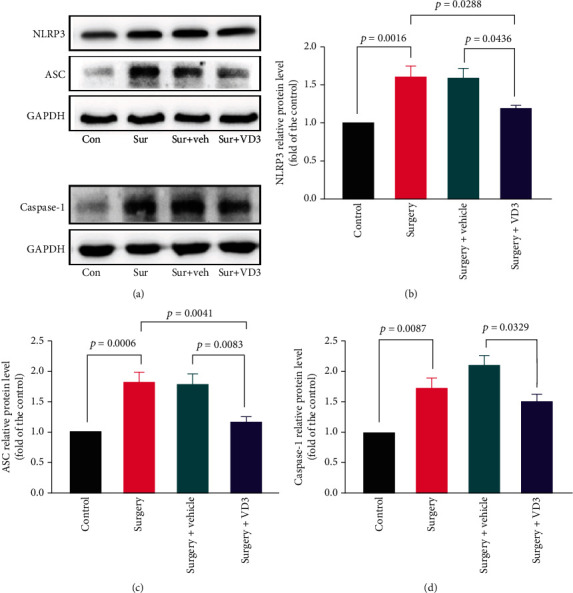
Expression levels of NLRP3, ASC, and caspase-1 in the hippocampus 24 hours after surgery. Western blot (a) and quantitative results (b–d) regarding the expression of NLRP3, ASC, and caspase-1. Data are presented as means ± SD (*n* = 5 per group).

According to our findings, tibial fracture surgery mice had higher levels of NLRP3 expression in the hippocampus than that of the control mice (*t* = 4.657, *P* < 0.01, Figures 4(a) and 4(b)). The levels of caspase-1 and ASC were upregulated after surgery (*t* = 3.839 and 5.144, both *P* < 0.01, Figures 4(a), 4(c), and 4(d)), which was reversed by VD3 pretreatment (*t* = 3.208 and 3.861, *P* < 0.05 and 0.01, Figures 4(a), 4(c), and 4(d)).

### 3.3. VD3 Decreased Postoperative Neuroinflammation in the Hippocampus of Aged Mice

**Figure 5:**
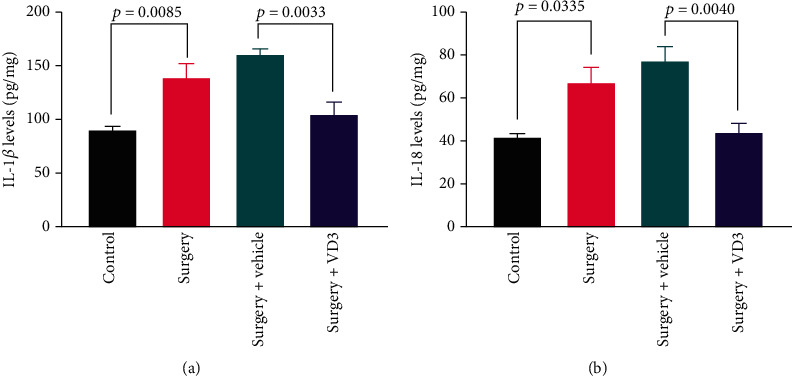
Hippocampal neuroinflammation following surgery. IL-18 and IL-1*β* protein expressions in the hippocampus. Data are presented as means ± SD (*n* = 6 per group).

Neuroinflammation is closely associated with POCD. Inflammatory cytokines were detected in the hippocampus 24 hours after surgery. IL-18 and IL-1*β* expressions in the hippocampus all increased after surgery (*t* = 3.105 and 3.701, *P* < 0.05, Figures 5(a) and 5(b)). Furthermore, VD3 inhibited the surgery-induced increase in IL-18 and IL-1*β* expressions in the hippocampus (*t* = 4.020 and 4.100, *P* < 0.05, Figures 5(a) and 5(b)).

### 3.4. VD3 Reduced the Hippocampal Postoperative Microglial Activation of Aged Mice

**Figure 6:**
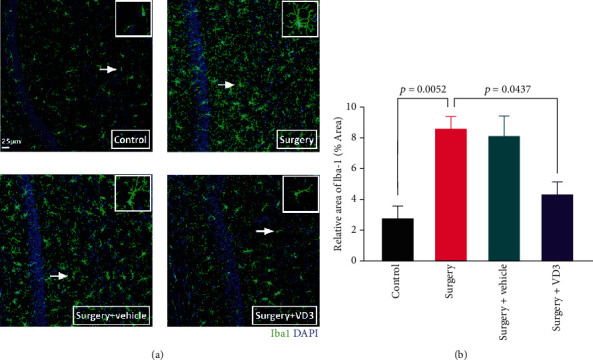
Microglial activation in the hippocampus following surgery. (a) Immunostaining for Iba-1 in the CA1 region of the hippocampus. The scale bar is 25 *μ*m. (b) Quantification of Iba-1-positive cells. Data are presented as means ± SD (*n* = 4 per group).

Due to the crucial role that microglia play in the emergence of neuroinflammation during central nervous system diseases, we sought to determine if microglial activation was present in the hippocampus 24 hours after surgery by employing immunostaining to identify the microglia marker Iba-1. There were more Iba-1-positive cells in the CA1 region of the mice in the surgery group than in the control group (*t* = 4.405, *P* < 0.01, Figure 6). In comparison to the surgery group, there were fewer Iba-1-positive cells in the VD3 group (*t* = 2.916, *P* < 0.01, Figure 6).

### 3.5. VD3 Treatment Prevents Oxidative Stress in the Hippocampus of Aged Mice

**Figure 7:**
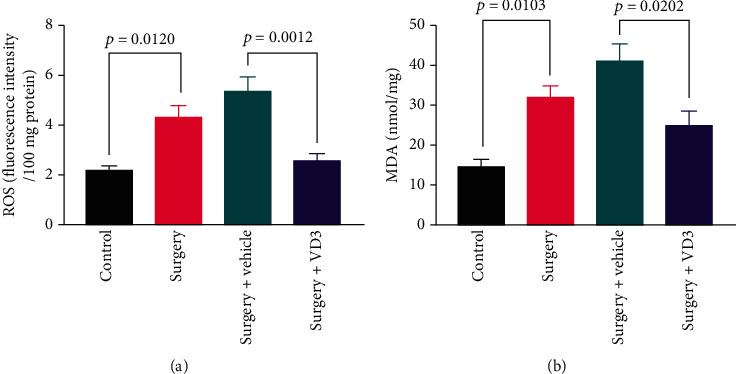
Expression levels of (a) ROS and (b) MDA in the hippocampus 24 hours after surgery. Data are presented as means ± SD (*n* = 5 per group).

According to the previous studies, oxidative stress affects hippocampal long-term potentiation, impairing cognition. We employed ROS and MDA to assess the status of oxidative stress. ROS and MDA levels in the hippocampus were significantly higher 24 hours after surgery compared to control mice (*t* = 3.687 and 3.759, both *P* < 0.05, Figure 7) but were significantly reduced by VD3 pretreatment (*t* = 4.744 and 3.439, *P* < 0.01 and 0.05, Figure 7).

## 4. Discussion

Our findings demonstrated that VD3 pretreatment significantly improved surgery-induced memory and cognitive dysfunctions in aged mice, which was linked to the inactivation of the NLRP3 inflammasome and the inhibition of neuroinflammation.

The neuroprotective, anti-inflammatory, and antioxidant properties of vitamin D have been widely studied in preclinical studies, which then led to the hypothesis that vitamin D could serve as a potentially modifiable risk factor for cognitive decline and neurodegenerative diseases [19–21], including the POCD [5, 8, 22]. However, preclinical research on the function of VD3 in the development of POCD is still limited. In this study, a tibial fracture surgery-based POCD model was employed, and the MWM test showed that pretreatment with VD3 dramatically decreased postoperative cognitive decline in aged mice. In models of LPS, hepatectomy, and sevoflurane anesthesia-induced cognitive dysfunction, protective benefits of VD3 supplementation on learning and memory were also noted [6–8].

Throughout the past decades, surgery-related cognitive impairment has been linked to neuroinflammation. A major factor in the development of cognitive impairment has been proposed to be the release of proinflammatory cytokines [18, 23, 24]. It has been demonstrated that the neuroinflammatory response during POCD pathophysiology is affected by variations in the levels of proinflammatory cytokines in the cerebrospinal fluid of postoperative patients. The overproduction of proinflammatory cytokines by activated microglia has also been demonstrated to contribute to chronic neuroinflammation [25, 26]. In the present study, it was determined that VD3 pretreatment suppressed the surgery-induced neuroinflammation by measuring the inflammatory cytokines in the hippocampus and the activation of microglia. Therefore, the anti-inflammatory activity of vitamin D3 may be a factor contributing to its protective benefits against POCD.

Vitamin D, whether vitamin D2 (ergocalciferol) or vitamin D3 (cholecalciferol), is delivered to the liver and converted to 25-hydroxyvitamin D (25(OH)D). The physiologically inactive molecule is subsequently transformed into 1,25-dihydroxy vitamin D (1,25(OH)_2_D), in the kidney or extrarenal tissues [27]. The blood-brain barrier is permeable to both 25(OH)D and 1,25(OH)_2_D. The 1,25(OH)_2_D signaling derived from 25(OH)D by 1-alpha-hydroxylase modulates brain activities by activating vitamin D receptors in neuronal and glial cells [28]. Vitamin D improves neurotransmitter imbalance by boosting acetylcholine synthesis and restoring dopamine balance in neurons [29, 30], as well as by decreasing the production of proinflammatory cytokines and upregulating anti-inflammatory cytokines [31, 32].

The NLRP3 inflammasome is an intracellular multiprotein complex that is a part of the innate immune system's inflammatory pathway [33]. It is composed of an apoptotic speck-like protein with a caspase recruitment domain, an inflammatory caspase, and a nucleotide-binding oligomerization domain-like receptor, which initiates the signaling cascade [33, 34]. The NLRP3 inflammasome regulates caspase-1 activation that controls the maturation and secretion of IL-1*β* and IL-18. Overproduction of IL-1*β* and IL-18 can result in chronic inflammation-related diseases, including POCD [9, 10]. By inactivating the NLRP3 inflammasome in preclinical models, VD3 could attenuate osteoarthritis, periodontitis, and cutaneous inflammation [15, 35, 36]. This study demonstrated that NLRP3 inflammasome was upregulated in the hippocampus of aged mice following tibial fracture surgery and that this process was inhibited by VD3 pretreatments. This finding indicated that suppression of the NLRP3 inflammasome may be associated with the protection of VD3 on cognitive function and the alleviation of neuroinflammation.

According to this study, pretreatment with VD3 significantly reduced the upregulation of ROS and MDA expressions in the hippocampus induced by surgical trauma. Recent research reveals that ROS is associated with cognitive impairment following surgery [37]. The overproduction of reactive oxygen species (ROS) is a critical upstream event that can activate NLRP3 inflammation [38]. In addition, VD3 has been shown to attenuate oxidative stress-mediated inflammation by inactivating the NLRP3 inflammasome [39]. Therefore, the anti-neuroinflammatory characteristics of VD3 might be attributable to the inactivation of the NLRP3 pathway, which was regulated by oxidative stress. But further research is needed to fully understand this hypothesis.

Although the NLRP3 inflammasome is being targeted as a therapeutic approach for the treatment of POCD, NLRP3 inflammasome inhibitors are not being used in the clinical setting [10]. According to this study, VD3 supplements can alleviate POCD symptoms by inhibiting NLRP3, providing an alternative to NLRP3 inflammasome-based POCD therapeutic strategies. The dosage of VD3 employed in this study was based on the previous research [8], and further research is necessary to determine the ideal dosage for treating POCD.

Models of dietary deficiency have been extensively used to study the behavioral consequences of altering vitamin D signaling on brain development. Dietary deficiency models may appear to have greater construct validity, but they are labor- and time-intensive, and their ability to interpret results is sometimes constrained [40]. In this study, aged mice were therefore supplemented with VD3 two weeks before surgery.

Due to its extremely low level and the lack of quantitative methods that can determine picomolar amounts, vitamin D content in brain tissue might be difficult to detect. Serum analysis is the most fundamental method for detecting vitamin D metabolites. The concentration of 25(OH)D, now thought to be the most abundant form of vitamin D in serum, may correctly represent the vitamin D levels [41].

It has been demonstrated that neuroinflammatory responses peak 24 hours after surgery [16, 17, 42]. Therefore, a time point of 24 hours following surgery was selected to assess neuroinflammatory variables. To rule out any potential confounding effects of behavioral testing on inflammatory markers, half of the animals in each group were sacrificed 24 hours following surgery for ELISA and immunostaining.

There are some limitations in this study: first, the upstream and downstream mechanisms of NLRP3 were not explored; second, only male mice were used, and gender differences in the effects of VD3 were not taken into account; third, VD3 was administered prophylactically; whether it has a therapeutic effect on POCD mice is unclear. Based on the findings of this study, the prophylactic and therapeutic use of medication can be studied along with the ideal dose of VD3. It is also worthwhile to investigate various administration techniques. In conclusion, our study showed that hippocampal inflammation and NLRP3 inflammasome activation were linked to tibial fracture surgery-induced cognitive impairment in aged mice, which could be alleviated by presupplementing with VD3. This finding provided a novel preventative strategy for clinically reducing postoperative cognitive impairment in elderly patients.

## Data Availability

The datasets used and/or analyzed during the current study are available from the corresponding author on reasonable request.
